# Rectal Mucinous Adenocarcinoma Invading Retrorectal Dermoid Cysts: A Case Report

**DOI:** 10.3389/fonc.2019.01389

**Published:** 2019-12-10

**Authors:** Rui Wang, Zhaopeng Yan

**Affiliations:** ^1^Department of Critical Care Medicine, Shengjing Hospital, China Medical University, Shenyang, China; ^2^Department of General Surgery, Shengjing Hospital, China Medical University, Shenyang, China

**Keywords:** rectal cancer, retrorectal cyst, tailgut cyst, developmental cyst, retrorectal space

## Abstract

Rectal mucinous adenocarcinoma is a subtype of colorectal adenocarcinoma, which is more aggressive and prone to invade adjacent normal organs or tissues compared with non-mucinous adenocarcinoma. Retrorectal dermoid cyst is a rare congenital disease, which usually are benign but with a potential for malignant degeneration. In this article, we report a case which presented a rectal mucinous adenocarcinoma invading into retrorectal dermoid cysts, indicating that besides adjacent normal organs or tissues, malignancies can also invade adjacent tumors, making their diagnosis and management more complicated. In such cases, double primary tumors should be considered, and they should be removed surgically.

## Introduction

The incidence of colorectal cancer ranks third among all malignancies worldwide, and rectal adenocarcinoma is the most common malignant tumor of the rectum (1, 2). The clinical practice guidelines for treatment of rectal adenocarcinoma include European ESMO guidelines and American NCCN guidelines. Retrorectal tumors are heterogeneous, including tailgut cysts, teratomas, epidermoid cysts, and dermoid cysts (3). As these are rare tumors in clinical practice, there are no guidelines to follow in the management of retrorectal tumors. Here, we describe a 50-year-old male patient, who presented with a rectal tumor complicated by retrorectal tumors. There was no clear border between the rectal tumor and the retrorectal tumors, and parts of the two tumors were fused together, which made the case difficult to diagnose and manage.

## Case Report

**Figure 1:**
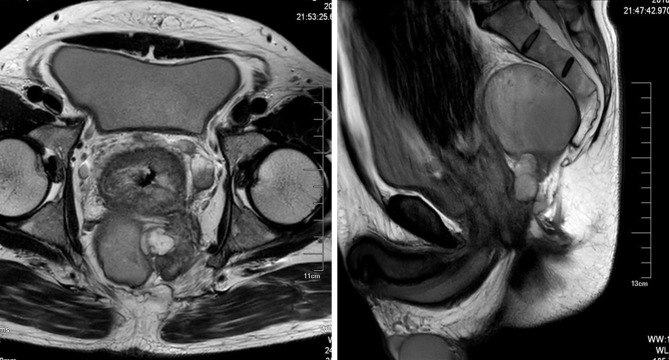
MRI: Several cysts were located in the retrorectal space, which had a clear border with the sacrum but adhered to the rectal tumor.

**Figure 2:**
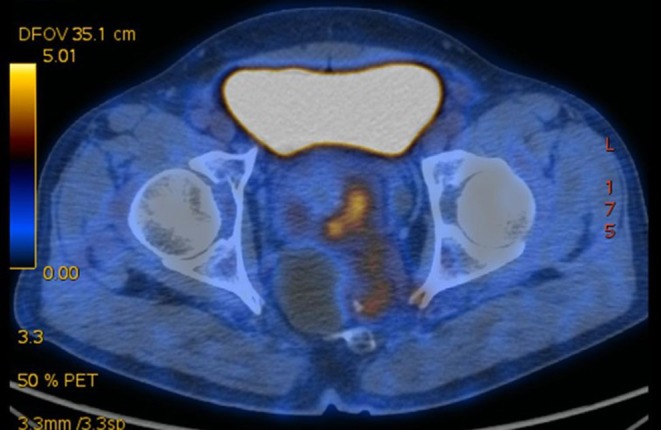
PET-CT revealed that the rectal tumor had high FDG accumulation; the cysts did not.

**Figure 3:**
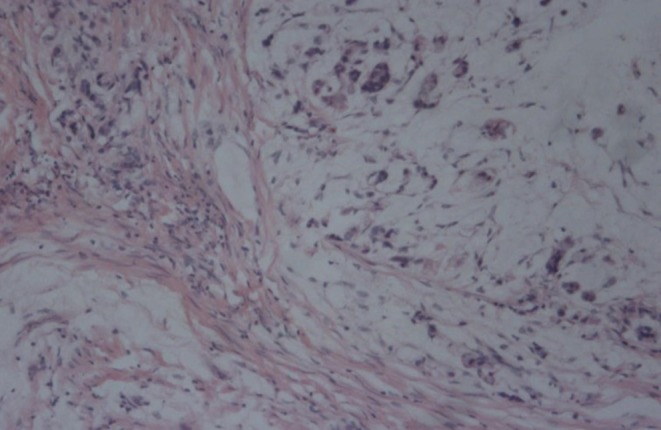
Histology revealed that the rectal tumor is a mucinous adenocarcinoma.

A 50-year-old previously healthy man presented to the emergency department with a 3-day history of worsening bloating, abdominal pain, and inability to pass flatus. The physician made a digital rectal examination and found a stricture of the rectum 2 cm above the anal edge. After depositing a tube into the proximal cavity of the narrowing part of the rectum, flatus and stool passed out, relieving the abdominal pain and bloating. A rectal MRI, an abdominal enhanced CT scan, and PET-CT were performed, finding that a rectal tumor growing circumferentially caused a stricture of the lower rectum. In the retrorectal space, several cysts were present, the largest of which had a diameter of about 8 cm. These cysts had a clear border with the sacrum but showed adhesion to the rectal cancer (Figure 1). The patient recalled that, 3 months previously, he received a routine health checkup, but a colonoscopy found nothing abnormal. The rectal tumor was probably missed

due to its location adjacent to the anus. CT and PET-CT revealed that the lower part of the rectum was thickened, and that accumulation of fluorine-labeled fluorodeoxyglucose (FDG), a marker for the uptake of glucose, was enhanced with an SUVmax of 6.36, indicating a suspicious malignancy. FDG accumulation in the retrorectal cysts was not enhanced (Figure 2). As the bowel was obstructed, the present case was a surgical emergency, and there was no opportunity to conduct a biopsy. MRI, CT, and PET-CT images revealed that the rectal lesion and retrorectal lesions were anatomically close. It was not clear if the case should be considered as double-primary disorders or as a single rare disorder (such as a rectal tumor secreting mucus that formed mucinous cysts or as retrorectal malignant tumors invading the rectum). Without a definite diagnosis, management was unclear. Because of the intestinal obstruction, however, and with the patient's consent, an abdominoperineal resection was performed. The postoperative pathology indicated that a rectal mucinous adenocarcinoma had invaded retrorectal dermoid cysts (Figure 3). The patient recovered well and, 1 month after the procedure, chemotherapy with the Xelox regimen (capecitabine and oxaliplatin) was initiated. He is now, 3 months later, being followed up.

## Discussion

Rectal adenocarcinoma is the most common malignant tumor of the rectum; other rectal tumors include squamous carcinomas, neuroendocrine tumors, stromal tumors, and lymphomas (4–7). These rectal tumors have various clinical and pathological features, including histology, biological behavior, and prognosis, requiring different management. Their treatment depends on a precise diagnosis and classification. For advanced rectal adenocarcinomas, neoadjuvant chemoradiotherapy is recommended before surgery (8–10). For tumors not sensitive to chemoradiotherapy, surgery is the first choice. For rectal lymphomas, chemotherapy without surgery can be considered (6–11).

Colorectal mucinous adenocarcinoma, a subtype of colorectal adenocarcinoma, accounts for 6–20% of all colorectal adenocarcinomas. The management of colorectal mucinous adenocarcinomas is not different from that of colorectal adenocarcinomas. Advanced mucinous adenocarcinoma, however, has a worse prognosis compared with non-mucinous adenocarcinoma (12, 13). Retrorectal dermoid cyst is a congenital disease, in the category of development cysts, which usually are benign but with a potential for malignant degeneration. About 30% of dermoid cysts are complicated by infection. Once the diagnosis of dermoid cysts has been made, surgical removal is indicated (3, 14, 15). For cases of dermoid cysts along with an adenocarcinoma, surgical removal of the cysts, and the adenocarcinoma may be the best treatment strategy.

## Conclusion

Malignancies have infiltrating potential, which means they can invade adjacent normal organs or tissues. This case presented a rectal mucinous adenocarcinoma invading into retrorectal dermoid cysts, indicating that malignancies can invade adjacent tumors, making their diagnosis and management complicated. In such cases, double primary tumors should be considered, and they should be removed surgically.

## Ethics Statement

Written informed consent was obtained from the participant for the publication of this case report.

## Author Contributions

ZY conceived and revised the manuscript. RW designed and wrote the manuscript.

### Conflict of Interest

The authors declare that the research was conducted in the absence of any commercial or financial relationships that could be construed as a potential conflict of interest.
